# Extracellular Metabolites from Industrial Microalgae and Their Biotechnological Potential

**DOI:** 10.3390/md14100191

**Published:** 2016-10-20

**Authors:** Lu Liu, Georg Pohnert, Dong Wei

**Affiliations:** 1 School of Food Science and Engineering, South China University of Technology, Wushan Rd. 381, Guangzhou 510641, China; luckyliulululu@163.com; 2 Institute for Inorganic and Analytical Chemistry, Bioorganic Analytics, Friedrich Schiller University Jena, Lessingstr. 8, Jena D-07743, Germany; Georg.pohnert@uni-jena.de

**Keywords:** microalgae, extracellular metabolites, exopolysaccharides, phytohormones, allelopathy

## Abstract

Industrial microalgae, as a big family of promising producers of renewable biomass feedstock, have been commercially exploited for functional food, living feed and feed additives, high-value chemicals in nutraceuticals, cosmeceuticals, and chemical reagents. Recently, microalgae have also been considered as a group that might play an important role in biofuel development and environmental protection. Almost all current products of industrial microalgae are derived from their biomass; however, large amounts of spent cell-free media are available from mass cultivation that is mostly unexploited. In this contribution we discuss that these media, which may contain a remarkable diversity of bioactive substances are worthy to be recovered for further use. Obviously, the extracellular metabolites from industrial microalgae have long been neglected in the development of production methods for valuable metabolites. With the advances in the last ten years, more and more structures and properties from extracellular metabolites have been identified, and the potential utilization over wide fields is attracting attention. Some of these extracellular metabolites can be potentially used as drugs, antioxidants, growth regulators or metal chelators. The purpose of this review is to provide an overview of the known extracellular metabolites from industrial microalgae which might be of commercial interest. The attention mainly focuses on the reports of extracellular bioactive metabolites and their potential application in biotechnology.

## 1. Introduction

Microalgae (including cyanobacteria) are unicellular or multicellular microorganisms that have successfully adapted to various environments. Autotrophic microalgae are photosynthetic, using solar energy, inorganic nutrients, and carbon dioxide (CO_2_) to produce proteins, carbohydrates, lipids and other valuable organic compounds while heterotrophic microalgae can be fermented using simple organic substrates (for example, glucose, acetate, glycerol) as carbon and energy sources for cell growth without light. Under these conditions high-cell-density biomass containing elevated amounts of valuable compounds such as fatty acids, pigments, proteins, vitamins, and active polysaccharides can be obtained [1]. So far, biomass-based production of industrial microalgae has been widely applied in the fields from food and feed to high-value chemicals for pharmaceutical and ecological applications [2,3,4]. In nutraceutical industries, *Arthrospira* (*Spirulina*) and *Chlorella* are the most important species in commercialization as health foods and nutrition supplements with various health benefits including enhancing immune system activity, anti-tumor effects, and animal growth promotion, due to their abundant proteins, vitamins, active polysaccharides, and other important compounds [5,6]. Microalgal carotenoids, with β-carotene from *Dunaliella* and astaxanthin from *Haematococcus* are commercially produced in large scale processes. Microalgal derived products are currently successfully developed for uses in cosmetics and pharmaceutical products [7,8]. Examples include the polysaccharides from cyanobacteria used in personal skin care products and extracts of *Chlorella* sp. which contain oligopeptides that can promote firmness of the skin [9]. In the pharmaceutical industries drug candidates with anti-inflammatory, anticancer, and anti-infective activities have been identified [10]. For instance, adenosine from *Phaeodactylum tricornutum*, can act as an anti-arrhythmic agent for the treatment of tachycardia and the green algal metabolite caulerpin is featured in studies of anti-tuberculos is activities [11,12]. Production of oleaginous microalgae are becoming attractive as alternative sources of biofuels with potential to meet global demand for renewable bioenergy [13]. Besides, microalgae such as *Isochrysis galbana, Nannochlor opsisoculata*, *Chaetoceros muelleri*, *Chaetoceros gracilis* and *P. tricornutum* have been long utilized in aquaculture as direct or indirect feed sources in hatchery to provide excellent nutritional conditions for early juveniles of farmed fish, shellfish, and shrimp [14,15].

In the past fifty years, large-scale cultivation of industrial microalgae has rapidly developed worldwide (Table 1). Most of the applications mentioned in Table 1 are successfully commercialized in different culture systems. It is known that a wide range of microalgae can release abundant extracellular bioactive compounds (proteins, polysaccharides, and other biopolymers, various small molecules) into the media [16,17]. Although the commercial cultivation of microalgae became increasingly popular, only algal biomass is processed to current products, while huge volumes of algae-free media are unexploited in flow through cultures and after biomass harvesting of batch cultures. Medium recycling to save culturing costs faces the big risk of growth inhibition. High volumes of spent media give rise to environmental pollution and cost of water and nutrition supply in cultivation when the media are discarded directly to the environment. Therefore the application of recycling methods motivated by the simultaneous generation of high value products from spent medium bears potential in commercial and environmental perspectives.

During the growth, microalgae produce and secrete metabolites such as acetate or glycerol into the medium [19]. Extracellular metabolites (EM) from microalgae have important ecological significances. For instance, marine microalgae release a large amount of dissolved organic substances (DOS), which serve as energy sources for heterotrophs in algal-bacterial symbiotic interactions [20]. Excretions into the pericellular space determine, to a great degree, the course of allelopathic interactions between microalgae and other microorganisms [21]. Some allelopathic compounds from microalgae are realized as environment-friendly herbicides or biocontrol agents with direct perspectives for their biotechnological use [22]. The enhanced oil recovery (EOR) using extracellular biopolymers from microalgae may be an upcoming field of application [23]. Moreover, some extracellular polysaccharides from microalgae have various bioactivities involving antitumor, anti-inflammatory, and antiviral activity, providing promising prospects for pharmaceutical applications [24]. Therefore, EM from microalgae might be potentially utilized in food, feed, cosmetics, and the oil industries, and be worthy to be developed for commercialization. In this review we present the first overview of the existing extracellular metabolites from industrial microalgae that might be of commercial interest. The attention mainly focuses on the reports of extracellular bioactive substances, with particular interests on their potential applications in biotechnology.

## 2. Exopolysaccharides (EPS)

Exopolysaccharides (EPS) comprise a group of important high-molecular-weight biopolymers that are secreted from microorganisms including microalgae into the surrounding environment during their growth or propagation [25,26]. They can either be loosely attached to the cell wall or excreted into the environment [27,28]. Many microalgae, especially a variety of red algae and cyanobacteria, are producers of structurally diverse EPS. EPS probably can protect cells from unfavorable stress in the natural environment [29]; additionally, EPS are involved in cell-to-cell interactions, adhesion, and biofilm formation [30,31]. EPS are widely used in the food industry as thickeners and gelling additives, which improve food quality and texture [32]. Currently, EPS have received much attention for their antibacterial, anti-oxidative, and anticancer properties, which lead to the development of promising pharmaceutical candidates [33,34]. Since EPS are released into the culture medium, they can be easily recovered and purified [35]. Different strategies used for the economical extraction and other downstream processing were discussed in a chapter of an excellent book [36]. Here, we focus on EPS with defined structures and bioactivities from industrial microalgae and their possible applications (Table 2).

### 2.1. Physiological Roles and Structures of EPS

The role of EPS in the physiology and interaction of microalgae is not completely understood, however several potential functions have been identified. In unfavorable conditions, microalgae produce high amounts of EPS for cell protection [48]. Polysaccharides may also be involved in the modulation of allelochemical activities [49]. Thus it was observed that an adaptive response of *Anabaena* PCC 7120, *Chlorella. vulgari*s, and other microalgae to microcystin toxins from crude extracts of *Microcystis aeruginosa* might be modulated by the production of both intra and extracellular polysaccharides [50,51]. Rhodophyte cells such as *Porphyridium* and *Rhodella*, are generously coated with polysaccharides. During growth, these polysaccharides partly dissolve into the medium. The common EPS from these types of microalgae are sulfated and contain different proportions of uronic acids. This is the reason that these EPS are negatively charged and this characteristic contributes to the anionic properties of the biopolymers [52]. For example, all polysaccharide fractions of the EPS from cell-free medium of *P. cruentum* contain sulfate and are mainly composed of galactose, glucose, xylose, and glucuronic acid [53]. Raposo et al. (2013) reviewed the sulfated polysaccharides synthesized by 120 marine microalgae, most of which are EPS. These heteropolymers consist mainly of galactose, glucose, and xylose in different proportions except those from *Gyrodinium impudicum*, which are homopolymers [16]. Most EPS from cyanobacteria are also complex anionic heteropolymers containing six to ten different monosaccharides, one or more uronic acids, and various functional substituents such as methyl, acetate, pyruvate, sulfate groups, and proteins [54]. For instance, the EPS from *Arthrospira platensis* are heteropolymer with protein (55%) moieties and a complex polysaccharide composition, containing seven neutral sugars: glucose, rhamnose, frucose, galactose, xylose, arabinose, and mannose, as well as two uronic acids, galacturonic acid and glucuronic acid [55].

*Dunaliella salina* is a unicellular green alga of outstanding halotolerance [56]. Salt stress induces the secretion of extracellular polymeric substances from *D. salina*. Mishra et al. (2015) speculated that the release of complex mixtures of macromolecular polyelectrolytes with high polysaccharide content contributes to the survival strategy of *D. salina* in varying salt concentrations. Four monosaccharides (galactose, glucose, xylose, and fructose) were detected in the hydrolysate of EPS from *D. salina* under salt stress [57,58]. In contrast, the water-soluble polysaccharides released by *Chlorella pyrenoidosa* contain galactose, arabinose, mannose, ribose, xylose, fucose, and rhamnose; their release depends on the cell photosynthetic activity and reproductive state [59]. Further studies are needed in order to fully unveil the functions of EPS from microalgae.

### 2.2. Bioactivities and Applications of EPS

Many studies have already highlighted the pharmacological activities of the EPS from red microalgae. The polysaccharides from *P. cruentum* exhibit antiviral activity against several kinds of viruses, such as Vesicular stomatitis virus (VSV), African swine fever virus (ASFV), and Vaccinia virus (VACV), as well as antibacterial activity against *Salmonella enteritidis* [37,60]. Moreover, the degraded products of the EPS from *P. cruentum* showed antitumor and immunomodulatory activities [38]. Besides *P. cruentum,* other *Porphyridium* species also exude EPS that exhibit hypocholesterolemic effects and can alter intestinal morphology in rats [39,40]. Crude extracellular polysaccharides of *Rhodella reticulata*, another species of single-celled red algae, exhibited free radical scavenging and antioxidant activity in a dose-dependent manner [41]. Besides EPS from red microalgae, the EPS from cyanobacteria, such as *Arthrospira platensis*, and the dinoflagellate *Gyrodiniumim pudicum* show also antiviral, antibacterial, and antioxidant pharmacological activities [44,47]. In addition, the sulfated EPS from *A. platensis* act as antithrombogenic, antiatherogenic, and anticoagulant. Their biological activity is related to molecular weight and the conformation in terms of solvent–polymer interactions [4].

As materials, the EPS of *P. cruentum* and *Rhodella maculata* showed outstanding drag-reducing properties at low concentrations, indicating their potential use as drag-reducing additives to increase the velocity of ships, thus reducing fuel consumption [61]. It is noted that some cyanobacteria EPS have rheological properties as flocculants [62]. Heteropolysaccharide EPS from *Nostoc flagelliforme* possess high intrinsic viscosity, excellent emulsification activity, and good flocculation capability [45]. Due to highly abundant negative charges, cyanobacterial EPS have been considered as promising metal immobilizing agents for the removal of positively charged heavy metal ions from effluents and wastewater. The cells from *Anabaena spiroides* have large polysaccharide capsules that continuously release EPS into the medium. Such EPS have the capacity to bind metals such as Cu(II), Pb(II), and Hg(II) [42]. They might thus be used to reduce the environmental concentrations of these heavy metals and lower their transfer into the food chain [63]. Additionally, the EPS of *Nostoc linckia* exhibit biosorption capacity of Co(II) and Cr(VI) ions, which depends on the contact time, pH, and initial metal ion concentration [43]. The EPS of *Chlorella stigmatophora* have a metal-complexing capacity of Zn^2+^ and Cd^2+^, which is possibly related to negative surface charges resulting from the presence of uronic acids or sulfate (or both) [46]. Microalgal EPS with metal-complexing capacity is of potential ecological importance and can be used as a natural metal chelate especially in the processes of biological water purification [42]. 

### 2.3. Strategies for EPS Yield-Increase

Although the EPS from microalgae have many potential applications, their low yield is one of the major limitations for scale-up in industry. The type and amount of EPS obtained from a certain microalgae-culture depends on several factors, such as culture system design, nutritional and culture conditions, as well as the recovery and purification process. Therefore, the configuration and optimization of production systems are critical for the further development of applications. Some strategies for promoting EPS yield are summarized in Table 3. In Bafanaa's study, an optimized medium was used to boost the EPS yield from *Chlamydomonas reinhardtii* strain RAC, which reached concentrations of 628 mg/L [35]. Díaz Bayona and co-workers (2000) examined the effect of nutritional conditions of *Botryococcus braunii* on EPS production by using two different culture media (BG11 and D medium). The higher salinity and nitrogen concentration in D medium contributed to the induction of EPS production [64]. EPS yield of *P. cruentum* was enhanced by the addition of sulfate and Mg^2+^ in the culture medium [59]. Interestingly, average increases of 33% in the EPS and 61% in the biomass production were achieved with *Chlorella* and *Spirulina* co-cultured with the Basidiomycete *Trametes versicolor* in submerged fermentation. This is one of the first examples, where the notion of altered physiological properties of microalgae under co-culturing conditions entered the field of aquaculture [65]. We suggest that co-cultures of microalgae and other microorganisms can be used more universally as a technology to increase the production of EPS, since microorganisms may respond to the interaction partners by secreting EPS as a strategy during unfavorable conditions [48]. Recently, a novel mutagenesis tool, atmospheric and room temperature plasma (ARTP) using helium radio-frequency glow discharge plasma jets, was effectively employed, leading to a high EPS-yield production of mutants of microalgae. In this case, ten mutants of *Crypthecodinium cohnii* exhibited enhanced EPS yield compared to the wild type strain. The best of mutants showed the maximum EPS volumetric yield of 1.02 g/L, which was 34% higher than that of the wild type strain [66]. 

## 3. Extracellular Proteins

### 3.1. Exoenzymes

Some scattered studies have demonstrated that the activity of extracellular enzymes in aquatic microbial ecology is of algal origin [67,68]. These exoenzymes released from microalgae include alkaline phosphatases, chitinases, β-d-glucosidases, proteases etc. and can influence the growth of microorganisms, chemical signaling, and biogeochemical cycling in ecosystems [69]. The study of these exoenzymes may help to optimize the nutrient supplement strategy in aquaculture. Nevertheless, only a few of the enzymes were isolated and purified. Further research is necessary in order to clarify the structure, bioactivity, and potential applications. Selected prominent enzyme classes are highlighted below [70].

#### 3.1.1. Extracellular Carbonic Anhydrases (eCA)

HCO_3_^−^ and CO_3_^2−^ are present in seawater as forms of inorganic carbon with concentrations greatly exceeding that of CO_2_. Microalgae have the ability to uptake HCO_3_^−^ directly or convert it into CO_2_ for consumption and subsequent photosynthetic fixation. Several microalgae (green algae, diatoms, dinoflagellates, and haptophytes) produce extracellular charbonic anhydrase (eCA) [71]. CA plays an important role in CO_2_ concentration mechanisms (CCM) [72]. In some microalgae, eCA enhances the uptake of CO_2_ from external HCO_3_^−^. The potential roles of these enzymes include CO_2_ supply, CO_2_ recovery, and pH regulation [73,74]. CA activity is impacted by CO_2_ concentration. For example, enrichment in CO_2_ can cause a decline of intracellular and extracellular CA activities in *C. pyrenoidosa* [75]. Additionally, the activity of eCA in *D. salina* could be affected by hypo-osmotic stresses. When exposed to salinities of 5‰–20‰, CA enzyme activities and expression levels were significantly inhibited by low salinity. As a result, photosynthetic carbon fixation inhibition occurred followed by slower algal growth [76]. However, compared to the intracellular CA, the nature, function, amount and activity of eCA is often poorly understood. Since the eCA may be critical to most algae to overcome the CO_2_ limitation and to increase the rate of photosynthesis and productivity, possibly, it can be used to aid inorganic carbon uptake [73]. Studies on potential biotechnological applications are scarce. 

#### 3.1.2. Extracellular Proteases

The green microalgae *Chlamydomonas coccoides* and *Dunaliella* sp. produce extracellular proteases. However, the report is very preliminary and failed to give details [77]. *Chlorella sphaerkii,* a unicellular marine chlorophyte, was also found to produce an extracellular protease. The capacity of this protease to cleave the substrate succinyl-l-Ala-l-Ala-Pro-l-Phe-4NA (Km = 4 μM at 37 °C, pH 8.6) was detected, with a considerable substrate specificity [77]. The diatom *Chaetoceros didymus* releases substantial amounts of proteases into the medium, this production is induced by the presence of the lytic bacterium *Kordia algicida* and is connected to the resistance of this alga against the effects of this bacterium [78]. Some proteases are of functional importance in viral life cycles, thus being attractive targets for drug development [79]. 

#### 3.1.3. Extracellular Phenoloxidases

Phenols are an important group of ecotoxins due to their toxicity and persistence [80]. Many microorganisms can degrade aromatic pollutants and use them as a source of energy [81], and the ability of microalgae to degrade a multitude of aromatic compounds including phenolic compounds is increasingly recognized. Some microalgae including *Chlamydomonas* sp., *Chlorella* sp., *Scenedesmus* sp. and *Anabaena* sp. are able to degrade various phenols such as pentachlorophenol, *p*-nitrophenol, and naphthalenesulphonic acids [82,83]. Though the metabolic degradation pathways are not fully understood, enzymes including phenoloxidase laccase (EC 1.10.3.2) and laccase-like enzymes are involved in the oxidation of aromatic substrates [81,84,85]. These exoenzymes can be potentially applied in the environmental degradation of phenolic pollutants.

### 3.2. Protease Inhibitors

An extracellular cysteine protease inhibitor, ECPI-2, was purified from the culture medium of *Chlorella* sp. The inhibitor had an inhibitory effect against the proteolytic activity of papain, ficin, and chymopapain. ECPI-2 contains 33.6% carbohydrate residues that may be responsible for the stability of the enzyme under neutral or acidic conditions. These inhibitor proteins from *Chlorella* may be synthesized to protect cells from attacks by e.g., viruses or herbivores [86]. Compared to organic compounds, peptide drugs are of relatively low toxicity to the human body. The development of peptide inhibitors as drugs is thus an attractive research topic in current medicinal chemistry [79]. Protease inhibitors are attractive agents in the treatment of specific diseases; for instance, elastase is of critical importance in diseases like lung emphysema, which motivates further investigation on microalgal protease inhibitors as valuable lead-structures in pharmaceutical development [87].

### 3.3. Phycoerythrin-Like Proteins

Phycobiliproteins are water soluble light-capturing proteins, produced by cyanobacteria, and several algae. These pigments have been explored as fluorescent tags, food coloring agents, cosmetics, and immunological diagnostic agents. Most of these pigments are synthesized and accumulated intracellularly. As an exception, the cyanobacteria *Oscillatoria* and *Scytonema* sp. release an extracellular phycoerythrin-like 250 kDa protein. This pigment inhibits the growth of the green algae *Chlorella fusca* and *Chlamydomonas* and can be potentially used as an algicide [88].

## 4. Organic Acids

### 4.1. l-Ascorbic Acid (AA)

l-ascorbic acid (AA), also known as vitamin C, is an essential dietary nutrient for humans, non-human primates, and a few other mammals. Currently, the main commercial production of AA is based on the Reichstein process which involves one bacterial fermentation and six chemical synthesis steps [89]. Several attempts have been undertaken to use the heterotrophic green microalgae *C. pyrenoidosa* for the production of AA, the highest content of intracellular AA reached more than 2% of cell dry weight [90]. Since the bulk of AA remained intracellularly, the process was patented as the method to produce AA-rich microalgae biomass which could be applied for animal feed or as dietary supplement. *Prototheca* sp., a group of acidophilic microalgae related to *Chlorella*, was suitable for extracellular AA production by fermentation due to its ability to grow well above pH 3.5, while maintaining AA productivity in an aerobic environment [91]. The fermentation resulted in extracellular accumulation of AA up to 76 mg/L, which is high enough to make the process economically feasible. Extracellular AA production could greatly reduce the manufacturing cost by eliminating the need of extraction from the cells. However, due to the rapid degradation of AA in high oxygen environments, this strategy is still under discussion [91]. 

### 4.2. Lactic Acid (LA)

Lactic acid (LA), an important molecule for food, pharmaceutical, leather and textile industries, is mainly produced through anaerobic fermentation by lactic acid bacteria (LAB) [92]. Currently, there is an increased demand of lactic acid for the synthesis of poly-lactic acid (PLA), which is a biodegradable bioplastic [93]. Some attempts have been made to employ microalgae as an alternative cell factory for fermentative LA production. A method for lactic acid production from lipid depleted *Nannochloropsis salina* biomass was developed, resulting in lactic acid yields up to 92.8% at sugar concentrations of 3–25 g/L [94]. However, the volumetric productivity is low and the method needs to be improved for cost effective production of lactic acid. Lactic acid was also found in the extracellular medium of some microalgal cultures. It predominated in the extracellular organic excretion of *Scenedesmus incrassatulus* grown in fresh and Black Sea water although the concentration was not reported [95]. Additionally, it was attempted to engineer a cyanobacterial cell factory for the production of lactic acid by integrating an *ldh* gene from *Bacillus subtilis* into the genome of a *Synechocystis* sp. strain. Promising results were obtained since lactic acid accumulated up to a several millimolar concentration in the medium [96]. Hence, metabolic engineering is a feasible approach for boosting LA biosynthesis in microalgae.

### 4.3. 5-Aminolevulinic Acid (ALA)

5-Aminolevulinic acid (ALA), which is a precursor of tetrapyrroles such as chlorophyll, haem, and vitamin B_12_, is used as a degradable insecticide and growth-promoting factor for plants [97]. Compared to the chemical synthesis of ALA, which requires many complex steps, biological production may be expected to be commercially viable. In the presence of levulinic acid (LA), a competitive inhibitor of ALA dehydratase in the medium, *C. vulgar* is produced about 1.5 mM ALA extracellularly. The synthesis of ALA in *Chlorella* may occur via the C-5 pathway, since its production was enhanced by the addition of glutamate, The Shemin pathway may also contribute to ALA production in the heterotrophic culture. Dark heterotrophic cultures may be better for ALA production than photosynthetic cultures of *C. vulgaris* since this species grows well aerobically in the dark and ALA synthesis was substantial [98,99]. However, no further study was found on fermentative ALA production by microalgae. 

### 4.4. Glycolic Acid

Glycolic acid, also known as hydroxyacetic acid, is one of the main metabolites of plants during the photorespiration process. It is also a raw material for organic synthesis in various areas including the textile industry, food processing and the pharmaceutical industry. For instance, glycolic acid is commonly used in skincare products. Glycolic acid is naturally produced by microalgae and can escape into the medium [100]. Though the glycolic acid production by microalgae has not yet been industrially exploited, some attempts have been made to improve the yield [93,94]. The release of glycolic acid from *Chlorella* cells was initially detected by Tolbert and Zill (1956), and the phenomenon was strictly dependent on light, aerobic conditions, and the presence of bicarbonate [100]. The concentration of glycolic acid in the culture media of the diatom *P. tricornutum* and the green alga *Tetraselmis gracilis* substantially increased when the cultures were aerated with nitrogen gas in comparison to atmospheric air. This might be caused by higher rates of photorespiration as CO_2_ was removed from the cultures [101].

## 5. Extracellular Lipids

The fact that oxygenic photosynthetic microalgae have the ability to accumulate abundant lipids intracellularly has been regularly exploited for the production of food additives and biofuel. Biodiesel generation from microalgal biomass is especially well researched since most oleaginous microalgae store easily accessible lipids in membranes and lipid droplets [102]. However, the whole process from harvesting, drying and cell disruption to lipid extraction is energy and time-consuming. Therefore, lipids-secreting microalgae are promising alternatives as producers of renewable biofuel.

### 5.1. Fatty Acids

Extracellular lipophilic substances from microalgae were targeted for the development of a consolidated bioprocess. Fatty acids (FAs) bound to glycerol, in the form of glycerol lipids can be used in biodiesel production. A golden brown chrysophyte *Ochromonas danica* secretes a mixture of free fatty acids (FFAs) into the medium, this mixture consists of a high proportion of polyunsaturated fatty acids, mainly C18:2*n*-6, C18:3*n*-3, and C20:4*n*-6. Those cells, grown under mixotrophic conditions, showed extracellular and intracellular FFAs production up to 20 and 51 mg/g cellular dry weight (CDW), respectively, higher than cells cultured under photoautotrophic conditions (up to 4 and 26 mg/g CDW, respectively) [103]. The fatty acid secretion of *O. danica* might be limited to the early and middle exponential growth phase. Lipids (polar lipids, sterols, free fatty acids, triacylglycerols and esters of sterols) were also observed in the medium of *Platymonas viridis* and *Nephrochloris salina,* two species that are candidates for use in mariculture. Their exolipids contain more saturated fatty acids compared to the intracellular lipids. The differences between endo- and exolipids might be caused by differential permeability and composition of the algal cell walls [104].

Mass spectrometry revealed around 1000 features in the outer cell wall secretions of the green microalga *Chlorella minutissima* UTEX 2341 grown on solid ATCC medium #5 (sporulation agar) in Petri dishes. Out of these fifty lipids, a series of triacylglycerols (TAG), sulfoquinovosyl diacylglycerols (SQDG), phosphatidylglycerols, and phosphatidylinositols as well as betaine lipids and diacylglyceryl-*N*,*N*,*N*-trimethyl-homoserines (DGTS), were annotated [105]. The main free fatty acids detected in the algal secretion were C16:0 and C18:0.

Salt stress induces an increase of extracellular lipids from *Scenedesmus* [106]. Chang et al. (2000) tried to create a fatty acid secretion platform in *Chlorella* by transforming target genes. Carrier proteins from *Synechocystis* sp. 6803 and *Arabidopsis thaliana*, which bind hydrophobic substances and move them through the cell membrane, significantly increased the fatty acid secretion in *Chlorella* [107,108].

### 5.2. Hydrocarbons

Among oleaginous microalgae, the colonial green alga *B. braunii* accumulates large quantities of hydrocarbons mainly in the extracellular space [109,110]. Cells of *B. braunii* associate via a unique colonial organization mediated by an extracellular matrix (ECM) mainly composed of long-chain polyacetal hydrocarbons which are cross-linked with hydrocarbons. It was proposed that *B. braunii* produces and secretes its hydrocarbons via an organized fenestrated endoplasmic reticulum (ER) system, and accumulates them in the hydrocarbon-based ECM [111]. Based on the chemical structure of hydrocarbons produced, *B. braunii* is classified into three principal races (A, B, and L). Among the three races, B secretes elevated amounts of cytoplasmic hydrocarbons. In contrast to race B, synthesis of extracellular hydrocarbons in race A is not completed in the cytoplasm, but occurs in extracellular space based on precursors exported from the cytoplasm [112]. The accumulation of extracellular lipids in *B. braunii* race B as well as race A is related to lipid bodies which are transformed from vacuoles [113].

Hiring microalgal cells as a “milking factory”, rather than growing the algae followed by harvesting the whole algal cells, may be a sustainable way to obtain the oils. There were some attempts on the repeated and non-destructive harvest of the external oil (hydrocarbons) from *B. braunii* [114,115]. Among several extraction solvents, heptane did not damage *B. braunii* if it was in contact for less than 20 min. In addition, a solvent-free method of gentle pressure (i.e., “blotting”) was introduced as alternative for external oil recovery by Moheimani et al. [109]. For the study of the long-term reliability of this oil recovery process, an integrated reactor was designed. In the authors view, immobilized biofilms may be the most realistic method for future scaling up of continued *Botryococcus* oil harvesting; in this way the dewatering stage, which is an energy consuming process of algae-to-biofuel production, can potentially be avoided [116].

## 6. Extracellular Phytohormones

Phytohormones are a group of signal molecules which play crucial roles in mediating growth, development and stress responses in plants [117]. Capacity to synthesize and release phytohormones including auxin, abscisic acid (ABA), and gibberellic acid (GA) has been well documented in higher plants. In macro and microalgae, occurrences of some endogenous substances that can act as phytohormones have been reported. Some of these metabolites are growth stimulating [118,119]. Besides, some externally applied synthetic regulators can affect the growth of microalgae. For example, 2,4-dichlorophenoxyacetic acid and kinetin significantly increase the growth of the chlorophytes *Haematococcus pluvialis* and *D. salina*. However, the metabolism and existence of extracellular hormones in algae is still poorly understood [120]. Knowledge about the metabolic and regulatory networks of microalgal phytohormones may provide fresh views and opportunities for microalgal cultivation and application [121].

### 6.1. Abscisic Acid (ABA)

Abscisic acid (ABA) produced by plants can act against diverse stress factors. Some microalgae and cyanobacteria produce extracellular ABA under different stress conditions. *C. vulgari*s and *Stichococcus hacillaris* increase extracellular ABA production under salt, acid or drought stress by 5–10 times [122]. More than 1 μg/L ABA was found in the culture medium after 6-days of the cyanobacteria *Nostoc muscorum*, *Trichormus versicolor* and *Synecbococcus leopolienssis* grown under salt stress. It is speculated that the extracellular ABA production should have an ecological significance, such as regulation of the association of microbes or increased resistance to stress factors [123]. However, the specific role of microalgal extracellular ABA is largely unknown. 

### 6.2. Indole-3-Acetic Acid (IAA)

During the last decades, endeavors were made to confirm microalgae as a source of the class of plant hormones auxins. Indole-3-acetic acid (IAA), the physiological activities of which relate to promoting cell division and elongation, is the most biologically active auxin. Evidences were provided for extracellular IAA production of some microalgae. IAA occurs in the medium of *C. pyrenoidosa* and *Scenedesmus armatus* at low concentrations [124]. Total indole released by *Chlorella minutissima* largely increased in the culture under continuous dark conditions with the addition of 5 g/L glucose for 48 h, which indicated endogenous hormones were affected by light regime and enrichment with glucose [125]. 

### 6.3. Gibberellic Acid (GA)

Gibberellic acid (GA) is a plant tetracyclic diterpenoid compound which stimulates the growth and development of plants. Although it remains uncertain whether microalgae are a source of GA, GA-like plant growth regulators were found in the cyanobacterium *Scytonema hofmanni* extracellular metabolites which demonstrated the ability to alleviate the salt stress effect on rice seedlings [126]. However, the presence of gibberellins still needs to be confirmed since the literature reports on microalgal extracellular gibberellins are scant. 

## 7. Allelopathic Chemicals

Chemically-mediated interactions between organisms occur throughout all the classes of microalgae. The term allelopathy is used for the interactions between organisms mediated by the release of biologically active chemicals into the environment. These compounds are often called allelochemicals or phytotoxins [127]. During the past decades, most studies involving allelopathy have focused on seaweeds and harmful algae, hoping to find novel ways towards seaweed management and red tide control [128,129]. Several species of microalgae can excrete remarkable biologically active metabolites into the medium, some of which are allelochemicals [130]. A better understanding of them will help to seek out a promising strategy to achieve the goal of producing bulk volumes of microalgae in large-scale cultivation [131]. The major types of allelochemicals from the medium of valuable microalgae are summarized briefly in Table 4.

### 7.1. Fatty Acids

A number of fatty acids may show inhibitory, stimulating or antifouling effects, and are assumed to be allelopathic chemicals. Chlorellin, which is attributed to the photooxidation products of unsaturated fatty acids, is an essential metabolite produced by *Chlorella,* causing inhibiting effects on the growth of *Chlorella pyrenoidosa* [136]. It was first found by Pratt et al. (1945) in cultures of *Chlorella*, where it accumulates over time in the medium when growth is relatively slow [137]. Chlorellinis is constituted of a mixture of C18 fatty acids (mainly stearic, oleic, linoleic, and linolenic acid), and its effects were observed during the co-culture of *C. vulgaris* and *Pseudokirchneriella subcapitata.* At low concentrations of chlorellin, a stimulation of growth was observed for both algae, while at higher concentrations inhibitory effects on both species were observed [132]. The authors suggest that the high prevalence of linoleic and linolenic acids in this mixture of fatty acids might contribute to the allelopathy. A growth inhibitor acting on eight species of microalgae including *D. salina* was successfully isolated and purified from filtrates of *Isochrysis galbana* [133]. *B. braunii* produces significantly larger amounts of free fatty acids compared to other phytoplankton isolates particularly α-linolenic and oleic acid. Chiang et al. (2000) speculated that such compounds could play roles as allelochemicals in favoring dominance of *B. braunii* in the natural environment by the isolation, identification, and verification of cell-free extract. However, there is still not enough evidence for extracellular free fatty acids to be responsible for allelopathy and for assigning to this phenomenon. Other effects of extracellular fatty acids include growth promotion or inhibition [138]. It was also reported that some long-chain unsaturated fatty acids have antimicrobial activities in low concentration and might be used in aquiculture [139].

### 7.2. Polyunsaturated Aldehydes

Polyunsaturated fatty acids can be broken down enzymatically by diverse lipoxygenase pathways to form polyunsaturated aldehydes, hydroxyl acids, and halogenated metabolites [140,141], and some polyunsaturated aldehydes have been made responsible for an allelopathic effect. These aldehydes are released upon cell damage in a wound activated process but can also be released into the environment by intact cells in the late stationary growth phase. The detection of polyunsaturated aldehydes in seawater supports this observation [21]. Oxylipins are involved in the hormonal regulation of mammals and plants, but are also well known for their olfactory properties, which makes them potential targets for the development of their industrial scale production.

### 7.3. Alkaloids

Alkaloids are frequently associated with allelopathy, especially in higher plants [142]. However, some metabolites of this class are also found in microalgae. The indolophenanthridine calothrixin A from the marine nitrogen-fixing cyanobacterium *Calothrix* sp. is an allelopathic compound able to kill several organisms and cell types such as bacteria and fungi. This compound inhibits DNA replication, RNA synthesis, and consequently protein synthesis in *Bacillus subtilis* [134]. Bromoanaindolone a brominated indole alkaloid isolated from the culture medium of the cyanobacterium *Anabaena constricta* may act as an allelopathic substance with outstanding antibacterial and anticyanobacterial activity as was verified by Volk et al. (2009) [136]. It was identified as 6-bromo-3-hydroxy-3-methyl-indol-2-one with a slight excess of the (3*R*) enantiomer [143].

### 7.4. Peptides

An excellent review published by Berry et al. (2000) reported that numerous cyanobacteria such as *Microcystis, Anabaena, Oscillatoria,* and *Nostoc* produce toxins with possible applications as algaecides, herbicides, and insecticides [17]. Among them, microcystins are cyclic heptapeptides with two variable amino acids and an unusual aromatic amino acid. For example, the microcystin-LR, produced and secreted out to the extracellular medium by *Anabaena flosaquae*, paralyzed the growth of the green alga *Chlamydomonas reinhardtii* [135].

### 7.5. Methanol

Microalgae can also be a source of methanol as was recently demonstrated during the investigation of a broad phylogenetic array of species [144]. Seven phytoplankton cultures were screened for their methanol production that reached 0.8–13.7 μM in the culture. Methanol per total cellular carbon was estimated in the range of 0.09%–0.3%, suggesting a potential use of microalgae for methanol production, once higher yielding isolates have been identified.

## 8. Summary and Prospects

In the past decades much progress has been made in acquiring knowledge of the extracellular metabolites from microalgae; however, the studies in this field are still fragmentary. Most investigations focused on the identification of global extracellular metabolites produced by a certain species under a given condition, and the biosynthetic pathways of these compounds remain largely unknown. Also, the transport mechanisms that are responsible for the release of these metabolites remain largely unexplored. An insight into the processes might provide key techniques for the manipulation of excretion levels. Recently, the use of exometabolomics increased the coverage of released metabolites thereby providing promising perspectives for the global investigation of algal responses to external stimuli [145]. These methods provide opportunities for the discovery of new extracellular products from microalgae. As discussed above, such metabolites are potential sources of new drugs, growth regulators, and other useful chemicals. Compared to the traditional microalgae biomass products, production of extracellular metabolites is not yet fully economically developed. Therefore the processes for the recovery and purification from media have to be optimized. In order to exploit those extracellular secretions it is crucial to gain deeper insights into the dynamics and stimulation of their excretion. In the near future, emerging tools including mainly metabolomic techniques and high-throughput bioassays will open new avenues for the exploitation of the not fully explored resource of extracellular microalgal metabolites. Further works on the development of technologies that succeed in increasing the yield and reducing the cost of commercial production of microalgal extracellular metabolites will ensure bright prospects for their applications in biotechnology.

## Figures and Tables

**Table 1:** Selected species of industrially applied microalgae, main products and application [3,16,18].

Species/Group	Product	Application Areas
*Spirulina* (*Arthrospira*)/Cyanophyta	Phycocyanin/allophycocyanin, biomass	Health food, cosmetics, feed additives
*Chlorella vulgaris*/Chlorophyta	Biomass, *Chlorella* growth factor (CGF), Chlorophyll	Health food, dietary supplement, feed additives
*Dunaliella salina*/Chlorophyta	Carotenoids (β-carotene)	Dietary supplement, cosmetics
*Haematococcus pluvialis*/Chlorophyta	Carotenoids (astaxanthin)	Health food, pharmaceuticals, feed additives
*Odontella aurita*/Bacillariophyta	Fatty acids (EPA, DHA)	Pharmaceuticals, cosmetics, baby food
*Porphyridium cruentum*/Rhodophyta	Polysaccharides, fatty acids (EPA, DHA), phycoerythrin	Pharmaceuticals, cosmetics, nutrition, thickener
*Porphyridium* sp./Rhodophyta	Phycoerythrin	Cosmetics
*Isochrysis galbana*/Haptota	Fatty acids (DHA)	Animal nutrition as living feed
*Phaedactylum tricornutum*/Bacillariophyta	Carotenoids (fucoxanthin), fatty acids (EPA)	Pharmaceuticals, cosmetics, animal nutrition as living feed
*Lyngbya majuscula*/Cyanobacteria	Immune modulators	Pharmaceuticals, nutrition
*Muriellopsis* sp./Chlorophyta	Carotenoids, lutein	Health food, food supplement, feed

**Table 2:** Species of microalgae producing EPS, bio-activities of the EPS and their possible applications.

Microalgae	Group	Bioactivities and Applications	References
*Porphyridium cruentum*	Rhodophytes	Antiviral, antibacterial activity Immunomodulation, Antitumor activities, Drag-reducing effect	[37,38,39]
*Porphyridium* sp.		Intestinal morphological modification, Hypocholesterolemic effect	[39,40]
*Rhodella reticulata*		Free radical scavenging, Antioxidant activity, Drag-reducing effect	[41]
*Anabaena spiroides*	Cyanophytes	Metal-binding Anti-thrombogenic, Antiatherogenic, anticoagulant, Antibacterial, antioxidant	[42] [4]
*Nostoc linckia*		Metal-binding	[43]
*Arthrospira platensis*		Antiviral, antibacterial, antioxidant	[44]
*Nostoc flagelliforme*		Emulsification, flocculation	[45]
*Chlorella stigmatophora* LB993	Chlorophytes	Metal-binding	[46]
*Gyrodinium impudicum*	Dinoflagellates	Antiviral	[47]

**Table 3:** Some cases of strategies for promoting EPS yield.

Species	EPS Yield (mg/L)	Technological Process	Reference
*Chlamydomonas reinhardtii*	628	One-at-a-time approach Plackett–Burman design Response surface methodology	[43]
*Botryococcus braunii*	44	Different medium comparison	[64]
*Phaeodactylum cruentum*	NA		[62]
*Chlorella vulgaris* *Arthrospira* (*S. platensis*)	7100 5420	Co-culture	[48]
*Crypthecodinium cohnii*	1020	ARTP	[66]

NA not available.

**Table 4:** Allelochemicals from different microalgal species.

Compounds	Microalgae	Effect	Target	Reference
Fatty acids				
Mixture of C18 fatty acids	*Chlorella vulgaris*	Growth stimulation Growth-inhibition	*Chlorella vulgaris* *Pseudokirchneriella subcapitata*	[132]
1-[hydroxyl-diethyl malonate]-isopropyl dodecenoic acid	*Isochrysis galbana*	Growth-inhibition	*Dunaliella salina* *Platymonas elliptica* *Nitzschia closterium* *Chaetoceros muelleri* *Chaetoceros gracilis* *Nitzschia closterium* *Chlorella minutissima* *Phaeodactylum tricornutum*	[133]
Polysaccharides	*Chlorella vulgaris* *Anabaena* PCC 7120	Toxin-resistance	*Microcystis aeruginosa*	[49]
Alkaloids				
Indolo phenanthridine calothrixin A	*Calothrix* sp.	Inhibitory effect on RNA and protein synthesis, DNA replication inhibition	*Bacillus subtilis.*	[134]
Peptides	*Anabaena flosaquae*	Inhibitory effect	*Chlamydomonas reinhardtii*	[135]

## Abbreviations

The following abbreviations are used in this manuscript:

EM	Extracellular Metabolites
DOS	Dissolved Organic Substance
EOR	Enhanced Oil Recovery
EPS	Exopolysaccharides
eCA	Extracellullar Carbonican Hydrase
CA	Carbonican Hydrase
VSV	Vesicular Stomatitis Virus
ASFV	African Swine Fever Virus
VACV	Vaccinia Virus
